# Recombinase-independent chromosomal rearrangements between dispersed inverted repeats in *Saccharomyces cerevisiae* meiosis

**DOI:** 10.1093/nar/gkad650

**Published:** 2023-08-07

**Authors:** Rachal M Allison, Dominic J Johnson, Matthew J Neale, Stephen Gray

**Affiliations:** Genome Damage and Stability Centre, University of Sussex, Falmer BN1 9RQ, UK; Genome Damage and Stability Centre, University of Sussex, Falmer BN1 9RQ, UK; Genome Damage and Stability Centre, University of Sussex, Falmer BN1 9RQ, UK; School of Life Sciences, Queen’s Medical Centre, University of Nottingham, Nottingham NG7 2UH, UK

## Abstract

DNA double-strand break (DSB) repair by homologous recombination (HR) uses a DNA template with similar sequence to restore genetic identity. Allelic DNA repair templates can be found on the sister chromatid or homologous chromosome. During meiotic recombination, DSBs preferentially repair from the homologous chromosome, with a proportion of HR events generating crossovers. Nevertheless, regions of similar DNA sequence exist throughout the genome, providing potential DNA repair templates. When DSB repair occurs at these non-allelic loci (termed ectopic recombination), chromosomal duplications, deletions and rearrangements can arise. Here, we characterize in detail ectopic recombination arising between a dispersed pair of inverted repeats in wild-type *Saccharomyces cerevisiae* at both a local and a chromosomal scale—the latter identified via gross chromosomal acentric and dicentric chromosome rearrangements. Mutation of the DNA damage checkpoint clamp loader Rad24 and the RecQ helicase Sgs1 causes an increase in ectopic recombination. Unexpectedly, additional mutation of the RecA orthologues Rad51 and Dmc1 alters—but does not abolish—the type of ectopic recombinants generated, revealing a novel class of inverted chromosomal rearrangement driven by the single-strand annealing pathway. These data provide important insights into the role of key DNA repair proteins in regulating DNA repair pathway and template choice during meiosis.

## Graphical Abstract

**Graphical Abstract:**
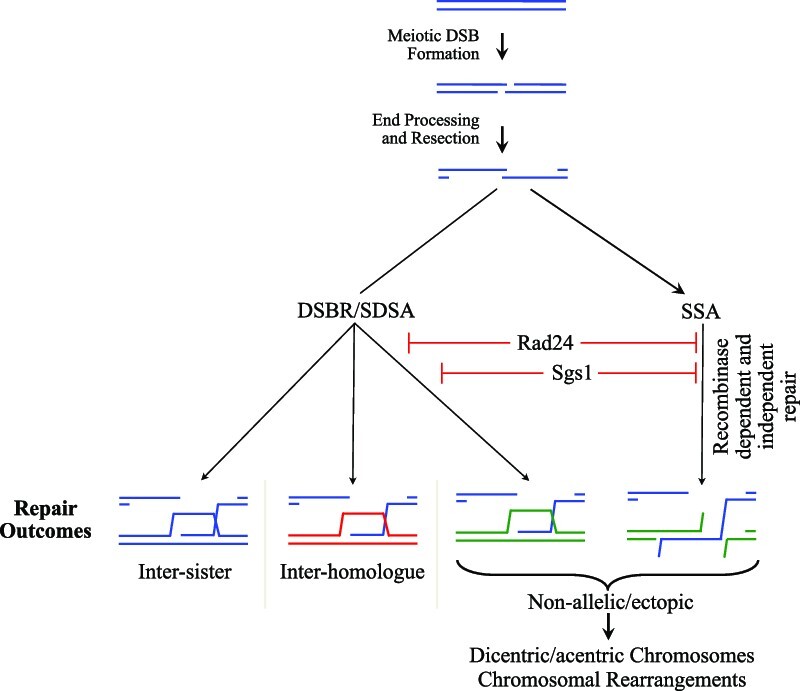

## INTRODUCTION

DNA damage repair mechanisms exist to ensure genome stability following the numerous endogenous and exogenous insults that the cell is exposed to daily. DNA double-strand breaks (DSBs), where both strands of the DNA helix are broken at the same locus, are particularly problematic. Failure to repair DSBs appropriately and accurately can lead to a range of mutation outcomes, from single-nucleotide changes to loss, gain and/or rearrangement of large chromosome regions. The two major pathways for repair of DSBs are non-homologous end joining (NHEJ) and homologous recombination (HR) (1). In NHEJ repair, both ends of the DSB are first processed by nucleases to remove DNA adducts or mismatches before being joined together by ligation, leaving the potential for small nucleotide deletions or additions (2). By contrast, during HR, DSB ends are resected generating a region of single-stranded DNA that invades a homologous template used for repair (1). Consequently, HR typically generates error-free repair, although inappropriate homologous template choice during HR can lead to loss or gain of genetic information, observed as unequal crossing-over events.

During meiosis, hundreds of DSBs are introduced in a programmed manner by the highly conserved topoisomerase-like protein Spo11, which becomes covalently attached to the DSB end during catalysis (3). In the budding yeast *Saccharomyces cerevisiae*, ∼140–200 DSBs form in every cell (4–8). Endonucleolytic removal of Spo11 occurs by MRX (Mre11–Rad50–Xrs2)/Sae2 processing, generating a short region of single-stranded DNA that is further extended by Exo1 (9–12). The single-stranded DNA generated favours repair by HR.

Formation of DSBs leads to the activation of DNA damage checkpoint machinery, preventing the cell cycle from progressing until DSB repair has occurred. Two major DNA damage checkpoint kinases function during meiosis, Mec1 and Tel1 (ATR and ATM orthologues, respectively) (13). In addition to their conventional cell cycle regulation role, both Mec1 and Tel1 regulate DSB formation and repair during meiosis (14–17). Specifically, Mec1/Tel1 phosphorylation of the HORMA domain-containing protein Hop1 instigates a repair bias from the homologous chromosome (18). In doing so, DSB repair during meiosis leads to not only gene conversion events by non-crossover repair from the homologue, but also reciprocal exchanges of genetic information between homologous chromosomes when crossovers occur. Consequently, allelic distribution varies within the haploid gametes.

HR is reliant upon homologous repair templates being available, and the action of RecA orthologues to undertake strand invasion. While it is not known what criterion defines a suitable template, *in vitro* data suggest that the meiosis-specific RecA orthologue Dmc1 allows for a more imperfect template to be used in repair, supporting the use of the homologous chromosome for repair, which may have allelic differences (19).

DNA sequences with full or partial homology to one another are found throughout genomes, enabling the potential for HR to take place between non-allelic sites, a process referred to as non-allelic homologous recombination (NAHR) or ectopic recombination. The insertion of the *LEU2* gene from strain X2180-1a at the *HIS4* locus in the *S. cerevisiae* SK1 strain background formed a meiotic DSB hotspot, enabling the investigation of DSB formation and repair dynamics (20). However, at the endogenous locus the *LEU2* gene was retained, albeit disrupted by insertion of the *Salmonella typhimurium hisG* gene, providing a perfect (or near-perfect) template for repair and the potential for ectopic recombination between *HIS4*::*LEU2* and *leu2*::*hisG*.

Analyses of Southern blotting at the *HIS4::LEU2* locus revealed the presence of ectopic recombinants in wild-type strains, a change in the type of ectopic recombinants formed in a *dmc1Δ* background and an increase in ectopic recombinant formation observed in DNA damage checkpoint mutants (15,21–23). Subsequent work revealed multiple pathways through which ectopic recombination can occur, with the RecQ helicase Sgs1 (24,25), synaptonemal complex protein Zip1, and recombinase and accessory proteins Rad51 and Tid1 functioning to suppress ectopic recombination (22,23). However, previous analyses have only investigated a subset of the ectopic recombinants that form from one side of the recombination hotspot studied and described only the local changes arising at that site.

To investigate ectopic HR further, we have probed for additional recombinants at local regions, chromosome-wide and under conditions that allow us to investigate inter-homologue/inter-sister bias in ectopic recombinant formation. Our data reveal novel ectopic recombinants, differing in frequency based upon the absence of DNA repair or checkpoint proteins, and the formation of acentric and dicentric gross chromosomal rearrangements, even under wild-type conditions. In addition, our results identify DNA repair proteins that function to reduce ectopic recombinant formation, not only by suppressing classic NAHR involving strand invasion, but also—unexpectedly—by suppression of the single-strand annealing (SSA) repair pathway, an outcome enabled via the coincident formation of Spo11 DSBs within high-frequency hotspots adjacent to repetitive DNA elements.

## MATERIALS AND METHODS

### Yeast strains

*Saccharomyces cerevisiae* strains (Supplementary Table S2) are isogenic to the SK1 subtype and were generated using standard genetic techniques. The base strain genotype is *ho*::*LYS2*, *lys2*, *ura3*, *arg4-nsp*, *leu2*::*hisG*, *his4X*::*LEU2*, *nuc1*::*LEU2*. *dmc1Δ*::*LEU2*, *dmc1Δ*::*hphMX*, *sae2Δ*::*kanMX6*, *rad51Δ*::*hisG-URA3-hisG* and *rad24Δ*::*hphMX* are all full replacements of the open reading frame with the selection marker. In the *sgs1-md* (*P_CLB2_-SGS1*) strains, the natural *SGS1* promoter is replaced with the *CLB2* promoter, whose activity is downregulated upon meiotic entry (26,27). In the *PRD1::pMN164* strains, there is a 7.1-kb plasmid insertion at the *PRD1* locus near the left telomere of chromosome III. pMN164 was created by cloning *PRD1* into pMN122/pRS306 at EcoRI–SmaI. The plasmid was linearized with BglII for integration at the natural *PRD1* locus by standard yeast transformation methods.

### Meiotic timecourses

Meiotic cultures were prepared as follows: YPD cultures (1% yeast extract, 2% peptone, 2% glucose) were diluted 100-fold into YPA (1% yeast extract, 2% peptone, 1% potassium acetate) and grown vigorously for 14 h at 30°C. Cells were collected by centrifugation, washed once in water, resuspended in an equal volume of prewarmed 2% potassium acetate containing diluted amino acid supplements (5 μg/ml of adenine, arginine, histidine, tryptophan and uracil, and 15 μg/ml leucine) and shaken vigorously at 30°C.

### DSB and ectopic recombinant analysis

**Figure 1.**
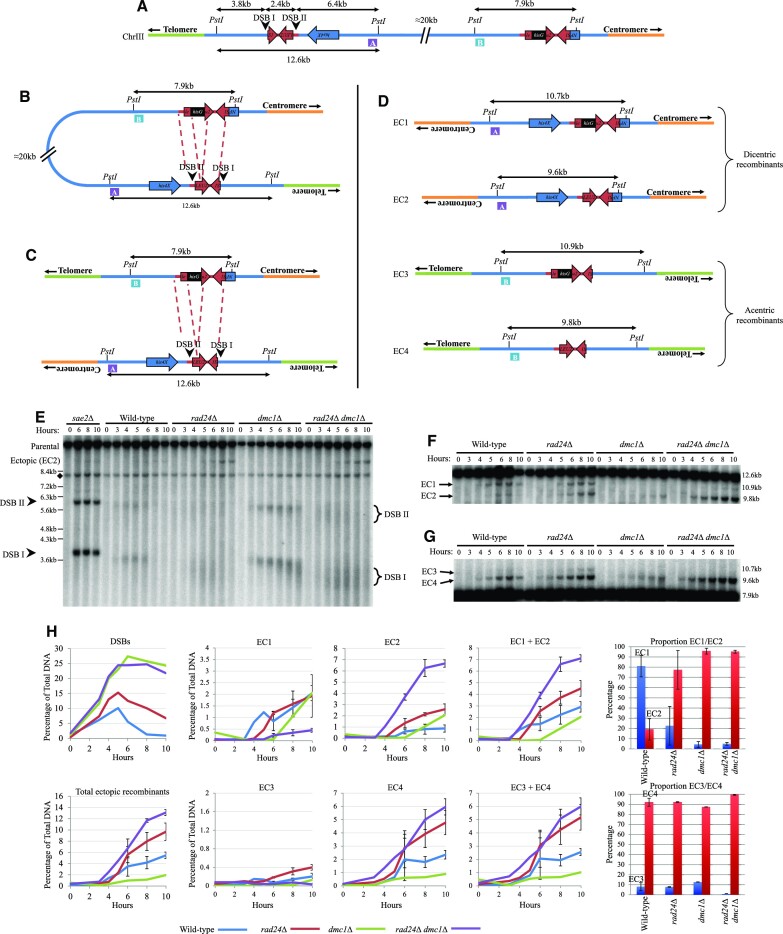
Ectopic recombinants form between *HIS4::LEU2* and *leu2::hisG* and are increased in *rad24Δ* backgrounds. (**A**) Schematic of the *HIS4::LEU2* and *leu2::hisG* loci of chromosome III of *S. cerevisiae*. Physical distances are displayed in addition to PstI restriction sites. DNA regions used as probes for Southern blotting are annotated as purple box A (*HIS4 LH* probe) and blue box B (*LEU2* probe). (**B**) Intra-chromatid and (**C**) inter-chromatid recombination between *HIS4::LEU2* and *leu2::hisG*. Red dotted lines indicate homologous sequences between loci. (**D**) Ectopic recombinant products generated from repair occurring between *HIS4::LEU2* and *leu2::hisG* with probes identifying recombinants indicated. EC1 and EC3 include *hisG* sequence in contrast to EC2 and EC4. Ectopic recombinant labelling is consistent throughout, but not with previous publications investigated due to inconsistencies existing from across articles. (**E**) Southern blot of the *HIS4::LEU2* locus using the *MXR2* probe previously described in (15). DSB signal is indicated by arrowheads and brackets. Non-specific signal is indicated by diamond. (**F**, **G**) Southern blots of ectopic recombinants using probes A (*HIS4 LH*) and B (*LEU2*), respectively. (**H**) Quantification of DSB signal from panel (E) and average ectopic recombinant signal from panels (F) and (G) and two additional repeats (blots not shown). Error bars plotting standard deviation are shown. Proportion of ectopic recombinant analysis from 10-h timepoint of the relevant strain with 95% confidence limits.

**Figure 2.**
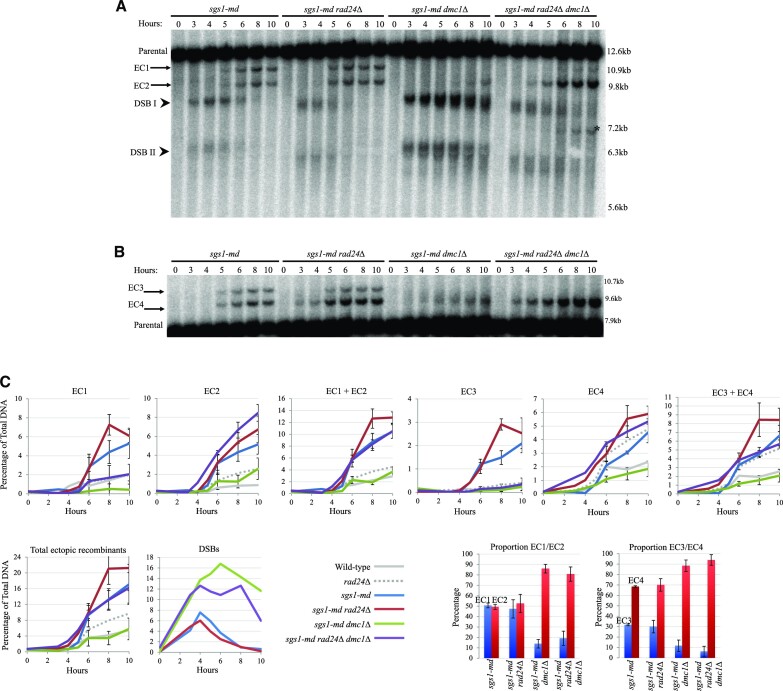
Ectopic recombinants are increased in *sgs1-md* mutants. Southern blots using (**A**) *HIS4 LH* probe (probe A) and (**B**) *LEU2* probe (probe B) showing DSBs at the *HIS4::LEU2* locus (arrowheads) and ectopic recombinants (arrows). Asterisk indicates additional ectopic recombinant likely forming between *HIS4::LEU2* and *nuc1::LEU2*. (**C**) Quantification of ectopic recombinant levels from panels (A) and (B) and two additional repeats (blots not shown). Error bars plotting standard deviation are shown. Wild-type and *rad24Δ* data are plotted from Figure 1. Proportion of ectopic recombinant analysis from 10-h timepoint of the relevant strain with 95% confidence limits.

DSB signals were detected using standard techniques by indirect end labelling of specific genomic loci after fractionation and transfer to Nylon membranes. For DSBs and ectopic products at *HIS4*::*LEU2*, genomic DNA was digested with PstI, separated on 0.7% agarose in 1× Tris–acetate–EDTA for ∼18 h at room temperature, transferred to Nylon membrane under denaturing conditions and then hybridized with a *HIS4 LH* probe (purple box, A) (Figures 1F, 2A and 5A). For DSBs at *leu2*::*hisG*, genomic DNA was digested with PstI, separated and transferred as above, and then hybridized with the *LEU2* probe (blue box, B) (Figures 1G, 2B, 5B and 6B).

**Figure 3.**
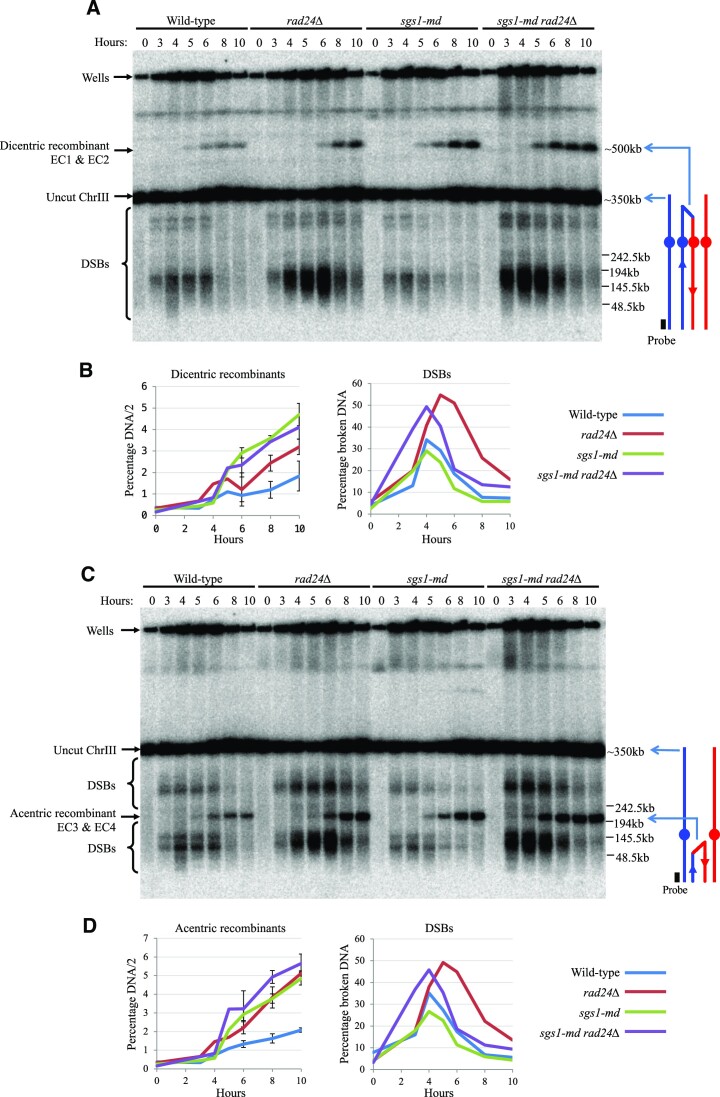
Ectopic recombination between *HIS4::LEU2 and leu2::hisG* creates acentric and dicentric molecules. Southern blots of chromosome III separated by PFGE and probed against (**A**) *GIT1* and (**C**) *CHA1* identifying dicentric and acentric molecules, respectively. (**B**, **D**) Quantification of ectopic recombinant signal and DSBs from panels (A) and (C) and two additional repeats (blots not shown). Error bars plotting standard deviation are shown.

**Figure 4.**
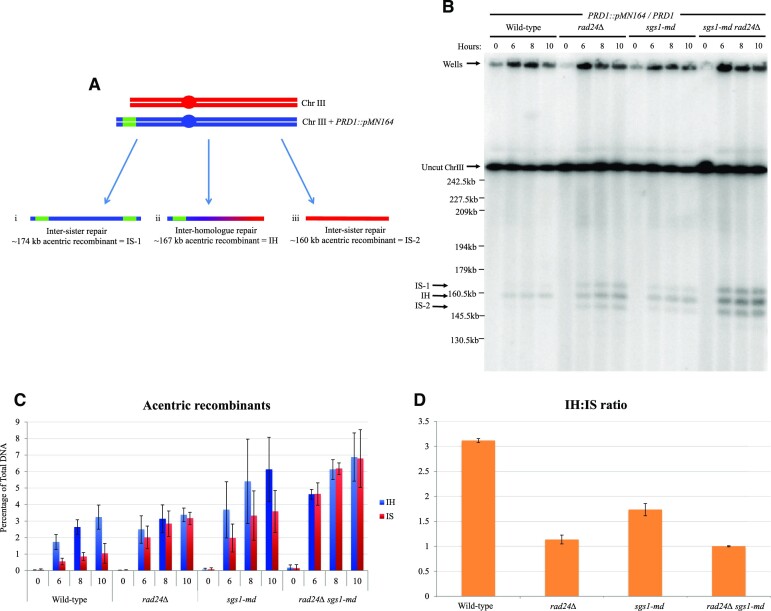
Inter-homologue/inter-sister acentric recombinant ratio is altered in *rad24Δ* and *sgs1-md* mutants. (**A**) Schematic of wild-type chromosome III and chromosome III increased in size due to insertion of *pMN164* at the *PRD1* locus. Inter-sister repair can lead to the formation of an acentric recombinant migrating at ∼174 kb (i) and ∼160 kb (iii) compared with an acentric recombinant forming by inter-homologue recombination of ∼167 kb (ii). (**B**) Southern blot showing chromosome III separated by PFGE and probed against *CHA1*. Acentric recombinants from inter-homologue and inter-sister repair are indicated with arrows. All strains are heterozygous for *PRD1* and *PRD1::pMN164*. (**C**) Quantification of acentric recombinants in mutant backgrounds from panel (B) and two additional repeats (blots not shown). Error bars plotting standard deviation are shown. (**D**) Ratio of inter-homologue to inter-sister acentric recombinant formation.

To quantify full-length acentric and dicentric chromosomes, chromosomal-length DNA was prepared after first immobilizing cells in agarose plugs as described (28). Chromosomes were separated using a CHEF-DR III pulsed-field gel electrophoresis (PFGE) system (Bio-Rad) using the following conditions: 1% agarose in 0.5× TBE; 14°C; 6 V/cm; switch angle 120°. For Figure 3, switch times were ramped from 12 to 20 s for 25 h. For Figure 4, switch times were ramped from 5 to 15 s for 40 h. After transfer to Nylon membrane under denaturing conditions, genomic DNA was hybridized with DNA probes that localize close to the left (*CHA1*) or right (*GIT1*) telomere of chromosome III. Radioactive signals were collected on phosphor screens, scanned with a Fuji FLA5100 and quantified using ImageGauge software (FujiFilm). When quantifying acentric and dicentric chromosomes, we divided the detected signal of these recombinants by 2, due to the presence of two copies of the probed sequence within the molecule to normalize signal relative to the rest of the lane. All DSBs and ectopic products are reported as a percentage of the total lane signal after background subtraction.

Values plotted with standard deviation bars are the mean of at least three independent timecourse experiments.

## RESULTS

### Ectopic recombination occurs between *HIS4::LEU2* and *leu2::hisG*

The natural *LEU2* locus is present on the left arm of chromosome III (S288c *YCL018W*, position 91324–92418). In strains used by many labs investigating meiotic recombination, this natural *LEU2* copy is interrupted by an ∼1.2 kb insertion of exogenous *hisG* DNA (Figure 1A) (20). In addition, ∼3.0 kb of sequence from the natural uninterrupted *LEU2* locus is often inserted to the left of the *HIS4* gene—an insertion that generates a pair of strong meiosis-specific Spo11 DSB sites, one at the inserted *LEU2* promoter (DSB II) and the other, stronger site, at the left-most insertion junction and coinciding with the fortuitous co-insertion of 72 bp of bacterial DNA (DSB I; Figure 1A) (20). Recombination between these *LEU2* repeats, intra-chromosomally (Figure 1B) or inter-chromosomally (Figure 1C), generates four possible ectopic recombination products (EC1–EC4; Figure 1D), two of which (EC1 and EC2) have been measured forming during meiosis using physical analyses (15,21–23,25).

EC1 and EC2 theoretically differ only by the presence and absence of the *hisG* sequence, respectively, as do EC3 and EC4 (Figure 1D). To determine whether this was the case, we generated strains with wild-type *LEU2*, without the *hisG* sequence, and observed both a general decrease in ectopic recombinant levels forming during meiosis and, importantly, a complete loss of EC1 and EC3, consistent with the latter classes of recombinant products forming between *HIS4::LEU2* and *leu2::hisG* and in a manner that retains the *hisG* sequence (Supplementary Figure S1).

During assembly of these diagrams, we realized that such ectopic recombinants are expected to produce acentric and dicentric rearrangements of chromosome III (Figure 1B–D), something not explicitly noted before (15,21–23,25). To assess the entire spectrum of ectopic recombination arising between the *LEU2* repeats, we designed restriction digests and DNA probes (Figure 1A–D) capable of assessing the relative frequency of all four ectopic products via Southern blotting of genomic DNA isolated from synchronous meiotic cultures of *S. cerevisiae*.

### Dmc1-independent ectopic recombination is increased in *rad24Δ* mutants

Previous work has demonstrated that the formation of two of the ectopic recombinants (labelled here as EC3 and EC4) was increased upon deletion of the *RAD24* DNA damage clamp loader (21), and intriguingly that the proportion of products is altered when the meiosis-specific RecA family recombinase, Dmc1, is absent (23). We corroborated these results, revealing an increase in EC3 and EC4 in *rad24Δ* cells, and a total loss of EC3 in the absence of Dmc1 (Figure 1E, F and H). Moreover, our analyses enabled us to also measure the frequency of EC1 and EC2. These products were also increased in *rad24* mutants, with formation of EC1 being highly dependent on Dmc1 activity (Figure 1G and H). The differential formation of EC1 and EC3, versus EC2 and EC4 in backgrounds with or without *DMC1*, respectively, led us to hypothesize that they may arise through mechanistically distinct DNA repair pathways (see below).

### Ectopic recombination is suppressed by Sgs1

Sgs1, the *S. cerevisiae* orthologue of the human BLM helicase, has been implicated at several steps in the meiotic recombination pathway (25,27,29–31). Sgs1 is most often termed as an anti-recombinase—promoting the dismantling of nascent recombination intermediates and homologous interactions (29,30). Sgs1 has previously been implicated in suppressing ectopic recombination (24,25), but it has been unclear in which pathways Sgs1 acts. Here, to help determine which ectopic recombination repair pathways Sgs1 is involved in, we assessed the frequency of ectopic recombination between the *LEU2* repeats in a yeast strain depleted for Sgs1 expression (*sgs1-md* ‘meiotic depletion’) in the presence and absence of Rad24 and Dmc1 (Figure 2).

Depletion of Sgs1 increased the frequency of all four ectopic products compared to wild-type cells, in most cases exceeding the levels observed in the *rad24Δ* single mutant (Figure 2A–C). Similar to *SGS1*^+^ cells (Figure 1F–H), loss of Dmc1 activity led to an overall reduction in ectopic repair efficiency in the *sgs1-md dmc1Δ* double mutant—coinciding with the expected accumulation of unrepaired DSBs (Figure 2A and C). However, as in the *dmc1Δ* single mutant (Figure 1F–H), the proportion of residual ectopic recombinants was shifted towards EC2 and EC4 (Figure 2A–C). This effect was further exacerbated in the *sgs1-md rad24Δdmc1Δ* triple mutant, which increased levels of ectopic recombinants 3-fold compared with the *sgs1-md dmc1Δ* mutant and had the heaviest skew towards EC2 and EC4 (Figure 2A–C). Additionally, a novel ectopic recombinant predicted to form between the *HIS4*::*LEU2* and *nuc1*::*LEU2* loci was detected in the *sgs1-md rad24Δ dmc1Δ* background (Figure 2A, asterisk, and Supplementary Figure S2). We conclude that, as for other types of homologous repair reactions, Sgs1 also appears to suppress the formation of ectopic recombination, as has been suggested elsewhere (24,25), and acts in both the Dmc1-dependent and -independent repair pathways.

### Ectopic recombination between *HIS4::LEU2* and *leu2::hisG* creates acentric and dicentric products

We noted that the inverted orientation of the homologous *LEU2* repeats means that ectopic recombination is expected to generate acentric and dicentric rearrangements of chromosome III—something that had not previously been documented (Figure 1B–D). To investigate this process, we used PFGE to separate whole chromosomes of *S. cerevisiae* undergoing synchronous meiosis, and sequentially hybridized the Southern blots using DNA probes to the two termini of chromosome III (Figure 3). We carried out these experiments in the *DMC1*^+^ background to avoid the complications in the quantification of ectopic products that would arise from most of the chromosome III-associated DSBs remaining unrepaired in the absence of Dmc1 activity.

To our surprise, bands migrating at the expected position for both acentric and dicentric chromosome III rearrangements were observed at significant levels (∼2% of total DNA) even in the wild-type control strain (Figure 3). Both acentric and dicentric products were increased 2–3-fold upon loss of either Rad24 or Sgs1 activity, with the double mutant displaying similar, or slightly greater, levels to the Sgs1 single mutant (up to ∼5–6% of total DNA) (Figure 3). These frequencies agree well with the frequencies of EC1 + EC2 and EC3 + EC4 measured earlier by standard Southern blotting (Figures 1 and 2), supporting the conclusion that they are independent readouts of the same molecular reactions.

### Ectopic recombination chromatid repair template choice is altered in *sgs1* and *rad24* mutants

Ectopic recombinants can form by repairing from the inter- or intra-sister chromatid or the homologous chromosome. To determine whether there is bias towards one repair template in ectopic recombination, we increased the length of one copy of chromosome III by insertion of a 7-kb plasmid on the left-hand end at the *PRD1* locus and measured acentric recombinant formation in diploid yeast heterozygous for the chromosome III variants (Figure 4A). In wild type, a total of ∼4% of acentric recombinants form, with a ratio of 3:1 bias towards repair from the homologous chromosome (Figure 4B–D). The inter-homologue ratio observed is similar to that reported for allelic inter-homologue DSB repair (32). In both *rad24Δ* and *sgs1-md* single and double mutants, the total frequency of acentric ectopic recombinants increases ∼2–3-fold compared to wild type, and inter-homologue bias decreases, with mutation of *rad24Δ* leading to little or no inter-homologue bias (Figure 4B–D). These results are consistent with the previous observation that the Mec1–Rad24 checkpoint pathway is required for establishment of the inter-homologue repair bias during allelic meiotic recombination (18). In addition, *sgs1-md* also lowered the inter-homologue ectopic recombinant ratio compared to wild type, consistent with previous observations that Sgs1 plays a role in establishing inter-homologue bias (24).

### Ectopic recombination is both Rad51- and Dmc1-independent in *rad24* mutants

The shift in ectopic product formation from EC1 to EC2 and from EC3 to EC4 that arises upon loss of Dmc1 activity led us to consider whether EC2 and EC4 products can arise from a novel—potentially recombinase-independent (i.e. Dmc1- and Rad51-independent)—molecular reaction.

**Figure 5.**
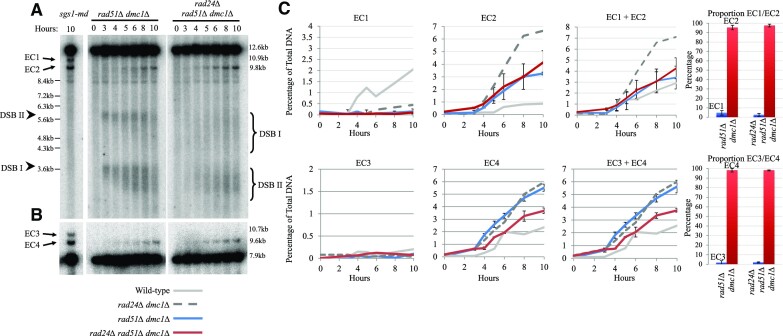
Ectopic recombinants form in *rad51Δ dmc1Δ* backgrounds. Southern blots using (**A**) *HIS4 LH* probe (probe A) and (**B**) *LEU2* probe (probe B) showing DSBs at the *HIS4::LEU2* locus (arrowheads) and ectopic recombinants (arrows). *Sgs1-md* 10-h meiotic timepoint included as a positional control for ectopic recombinant migration. (**C**) Quantification of ectopic recombinant levels from panels (A) and (B) and two additional repeats (blots not shown). Error bars plotting standard deviation are shown. Wild-type and *rad24Δ dmc1Δ* data are plotted from Figure 1. Proportion of ectopic recombinant analysis from 10-h timepoint of the relevant strain with 95% confidence limits.

Before taking this further, we first tested the requirement of Rad51 in the formation of the four ectopic recombinants (Figure 5). Consistent with prior observations made in single *dmc1Δ* (Figure 1) or single *rad51* mutants (21,23), we observed that the shift towards EC2 and EC4 that arises in *dmc1Δ* mutants was also independent of Rad51 (Figure 5)—indicating that these ectopic products were likely to be forming without a requirement for the canonical DNA strand invasion and homology recognition steps usually associated with homology-directed repair.

The main homology-directed repair process that is independent of RecA orthologue activity is SSA (33). SSA is capable of stitching together a pair of repeats—deleting the intervening region—in response to the two regions becoming single-stranded. In genetic assays, this reaction most often involves bidirectional resection from a single DSB that forms between a pair of direct repeats [e.g. (34,35)]. However, importantly, resection from a single DSB is unable to create single-stranded DNA with the required complementary polarity to enable the annealing of a pair of inverted repeats. Moreover, the *LEU2* inverted repeats used in our assay are >25 kb apart, significantly farther than meiotic resection has been measured to traverse (11,12).

### *leu2::hisG* is a frequently breaking meiotic DSB hotspot and provides homology for repair by SSA

**Figure 6.**
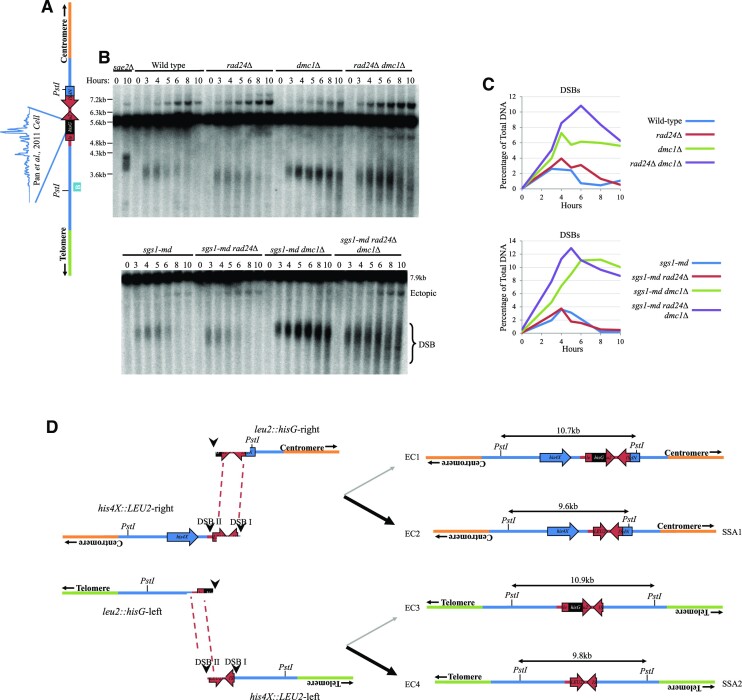
*leu2::hisG* is a frequently breaking meiotic hotspot and provides homology for SSA. (**A**) Schematic of the *leu2::hisG* locus with Spo11 oligo levels from (5) annotated. (**B**) Southern blot using *LEU2* probe (probe B, blue box) showing DSBs are the *leu2::hisG* locus. (**C**) Quantification of DSBs from panel (B). (**D**) Ectopic recombinants formed by SSA between *HIS4::LEU2* and *leu2::hisG*.

Given that DSBs arising within the *HIS4*::*LEU2* locus would be incapable—alone—of enabling an SSA repair reaction involving *leu2*::*hisG*, we sought to assess the frequency of DSBs forming across the *leu2*::*hisG* region (Figure 6). Previous whole-genome analysis of Spo11 DSB formation indicated that a significant number of reads mapped to within the exogenous *hisG* insertion (5). To confirm this observation, we used Southern blotting to probe the *leu2*::*hisG* locus in meiotic genomic DNA prepared from a *sae2Δ* strain where DSBs accumulate without repair and also in the set of strains used in our ectopic analyses (Figure 6B). Consistent with the whole-genome mapping data, we detected a strong meiosis-specific DSB site located within the *hisG* insertion (strength ∼10% of total DNA, comparable to other strong sites like *ARE1*), and consistent with the genome-wide mapping data (Figure 6C) (5).

We then explored the possible outcomes for SSA-mediated repair of two DSBs—one arising at *hisG* and the other at either of the two hotspots within the *HIS4*::*LEU2* insertion (Figure 6D). Due to the location of homologies relative to the major DSB sites, we predict there to be only two major products possible via a process of SSA—both of which are expected to lack the *hisG* sequence itself because this will be trimmed away in the 3′ flap removal stage of SSA. SSA product 1 involves repair between *LEU2* homology to the right of *hisG* and *LEU2* homology to the right of *HIS4*::*LEU2* DSB I. Because both homologies are centromere-proximal, the expected product is a dicentric identical in structure to EC2. By contrast, SSA product 2 involves repair between *LEU2* homology to the left of *hisG* and *LEU2* homology to the left of DSB II. The expected product in this case is an acentric product identical in structure to EC4.

Taken together, these analyses strongly suggest that SSA is another—previously unconsidered—mechanism that can generate gross chromosomal rearrangements and deletions during meiosis between dispersed genetic elements. While a less than ideal repair outcome, the potential to form ectopic recombinants by SSA is likely more favourable than unrepaired DSBs, which would provide a greater potential for chromosomal loss or gain during chromosome segregation. Moreover, the fact that ectopic recombination outcome skews strongly towards those compatible with SSA when Dmc1 recombination activity is abrogated suggests a hitherto unforeseen role for the canonical strand invasion-dependent HR pathway in suppressing deleterious outcomes—something that may be especially important in meiotic cells that experience a high frequency of DSBs forming and repairing across the genome within the same window of time (see the next section for further details).

## DISCUSSION

Previous investigations of ectopic recombination during *S. cerevisiae* meiosis have focused on two of the four products generated between the *HIS4*::*LEU2* and *leu2*::*hisG* loci. Our analysis has investigated the mechanisms involved and characterizes in detail all ectopic recombination outcomes at the local and chromosomal scales.

**Figure 7.**
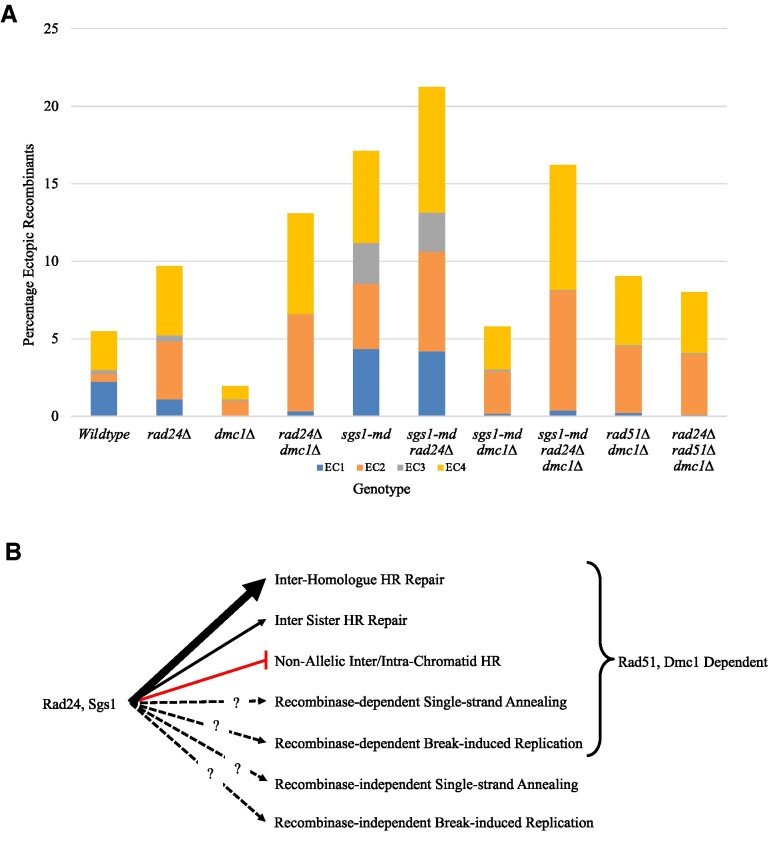
Ectopic recombinant levels and repair pathways. (**A**) Average ectopic recombinant levels from each genotype at 10 h from Figures 1, 2 and 5. Bars depict the proportion of each ectopic recombinant. (**B**) Mechanisms of DSB repair during meiosis. Rad24 and Sgs1 function to promote inter-homologue HR repair and inhibit inter- and intra-chromatid HR. DSB repair can occur via recombinase-dependent and -independent SSA and break-induced replication (BIR), but the relationship between Rad24 and Sgs1 regulation is unknown.

Consistent with previously published data, our data show that mutation of Rad24 and Sgs1 increases ectopic recombination (Figure 7) (15,21–25). Interestingly, our data reveal that when combined with *dmc1Δ*, the types and levels of ectopic recombinants that form change (summarized in Supplementary Table S1). Using a strain lacking the *hisG* insertion at *LEU2*, we revealed not only a loss of ectopic recombinants that contain the *hisG* sequence (EC1 and EC3), but also a general decrease in ectopic recombinant levels, suggesting that the main pathway for ectopic recombinant formation depends on the *hisG* insertion—itself a strong meiotic DSB hotspot. The levels of ectopic recombinants observed in the *rad24Δ dmc1Δ*, *sgs1-md dmc1Δ* and their respective single mutants lead us to conclude that inhibition of an ectopic repair mechanism is occurring in the *sgs1-md dmc1Δ* background, which is not present in the *rad24Δ dmc1Δ* background. Because an increase in ectopic recombination is observed when removing Rad24 function from the *sgs1-md dmc1Δ* strain, we propose that it is Rad24 that functions to inhibit ectopic recombinant formation in this strain. The mechanism by which Rad24 inhibits ectopic recombination is unclear, but may be related to either its checkpoint role or the increased resection observed in Rad24 mutants (summarized in Supplementary Table S1) (36,37).

The DNA checkpoint clamp loader Rad24 and the helicase Sgs1 are known to function throughout meiotic recombination (15,22–24,27,29,30,36–38). As such, Rad24 and Sgs1 aid in defining repair template choice, both allelic and non-allelic, and, as our data show, sister chromatid and homologous chromosome choice (Figure 4) (18). Given the previously described actions of Rad24 and Sgs1, one model for preventing ectopic recombination by these proteins may be that Rad24 functions early, defining correct template choice, and Sgs1 functions later to unwind ectopic strand invasion events. Layered on top of these mechanisms are the activities of Rad51 and Dmc1 that promote HR repair, enabling DNA invasion at homologous templates but also tolerating the allelic variation that may exist between homologs. However, while this model seems appealing, the decrease in inter-homologue bias observed in the *sgs1-md* background provides further insight into Sgs1 activity in regulating ectopic recombinant formation at an early stage.

Previous analysis of inter-homologue repair at *HIS4*::*LEU2* relies upon detecting differentially sized DNA molecules generated by recombination between chromosomes containing restriction site polymorphisms. However, non-crossover repair products generated via inter-sister repair products cannot be distinguished from the parental DNA. Given that all ectopic crossover recombinants formed lead to a product of a different size compared to the parental chromosomes, using two differentially sized chromosome III alleles allowed us to distinguish all acentric molecules and determine the ratio of inter-sister to inter-homologue template use. In doing so, we have revealed that in the *rad24Δ* background ectopic recombinants form at similar levels between sister chromatid and homologous chromosome. Strikingly, we show that *sgs1-md* also has a reduction in inter-homologue bias in ectopic recombinant formation. Previous observations have defined Sgs1 as playing a role in disassembling D-loop formation, promoting synthesis-dependent strand annealing (SDSA) and class I crossover repair (24,27,29,30). The results presented here, and consistent with published data, support a role of Sgs1 in promoting inter-homologue repair (24).

Prior work has shown that ectopic recombination does not require Ndt80 expression, a transcription factor that regulates expression of middle meiosis genes, some of which are required for Holliday junction resolution (21). Given this, ectopic recombinants may be generated by the activity of Ndt80-independent structure-specific nucleases, such as Mus81, long-range SDSA, BIR or SSA, although we cannot rule out canonical Holliday junction resolution mechanisms also playing a role. In our analysis, mutation of *DMC1* decreases the frequency of ectopic recombinants formed that retain the artificially inserted *hisG* sequence (EC1 and EC3) with the remaining ectopic recombinants (EC2 and EC4) still forming in the absence of both *RAD51* and *DMC1* (summarized in Supplementary Table S1 and Figure 7). These results support the idea that ectopic recombinants formed in the *dmc1Δ/rad51Δ dmc1Δ* background are generated by a strand invasion-independent mechanism, therefore ruling out SDSA as a potential repair mechanism. We propose that the remaining ectopic recombinants form by SSA (see below) due to the otherwise extensive (chromosome-length) DNA synthesis that would be required to generate ectopic products by Rad51/Dmc1-independent BIR. On this point, we further note that acentric and dicentric gross chromosomal rearrangements arise with similar kinetics (Figure 3) despite, if they were forming via BIR, the latter requiring the generation of ∼10 times the amount of newly synthesized DNA. Although further investigations to unambiguously elucidate the repair mechanism are theoretically possible, such experiments are technically challenging due to the low cell viability of the multiple mutant strain required to test this question [a DNA repair mutant (*rad24Δ* or *sgs1-md*) in a recombinase-defective background (*rad51Δ dmc1Δ*) in combination with a BIR mutant or SSA mutant].

Combined, our data lead us to conclude that the SSA repair pathway is utilized in recombinase-defective backgrounds. Interestingly, it is only through insertion of the bacterial *hisG* sequence, leading to the formation of a second strong Spo11 DSB hotspot, that the formation of single-stranded DNA homology required for repair by SSA takes place. Thus, we infer that ectopic SSA products arise through the coincident formation of Spo11 DSBs at both *HIS4*::*LEU2* and *leu2*::*hisG*, as has been demonstrated to be possible (14), and consistent with the reduction in ectopic recombinants that occurs when the *hisG* insert is removed (Supplementary Figure S1).

While the ectopic repair characterized in this study occurs via the *LEU2* repeats adjacent to the Spo11 hotspots described, it should be noted that insertion of *hisG* sequence has been used widely to disrupt gene function in meiotic SK1 *S. cerevisiae* strains (observed in strains defective in the HO endonuclease, *his3* gene and *trp1* gene, for example) and, as such, is likely to have introduced novel Spo11 DSB hotspots within each ectopic *hisG* repeat. Consequently, the *hisG* insertion may have altered the local recombination landscape, enabling ectopic recombination between dispersed *hisG* repeats via HR and SSA repair pathways.

Mutation of DNA repair proteins also contributes to other factors that may influence ectopic recombination (summarized in Supplementary Table S1). For example, mutations of Rad24 and Rad51/Dmc1 are known to increase resection lengths (37,39,40) and *rad24Δ* also increases the preference for Spo11 DSBs to arise at DSB1 versus DSB2 within the *HIS4*::*LEU2* hotspot (15). Due to the location of the DNA homologies involved, we would predict that increased resection tract lengths would favour EC1 and EC3 products, whereas an increased bias of DSB1 over DSB2 would promote the formation of EC1 and EC2. While it is likely that these effects have an impact on the outcome of ectopic recombination, the ectopic recombinants formed in these backgrounds do not fit these predictions. Therefore, our data suggest that the absence of these DNA repair proteins directly alters DSB repair pathway choice, and that this has a dominant role in the amount and type of ectopic recombinants formed.

Taken together, our data identify differences in ectopic recombinants that form across mutant backgrounds originating through different DSB repair pathways. For the first time, we characterize the formation of large chromosomal translocations arising by ectopic recombination in *S. cerevisiae* meiosis, leading to the generation of acentric and dicentric chromosome fragments. Such rearrangements affect copy number variation across the genomic region and dicentric chromosomes will cause chromosome segregation problems, affecting cell survival. While we have identified these specific ectopic recombination outcomes, we recognize that gaining greater understanding of the different types of recombinants and rearrangements that form across wild-type and mutant backgrounds both within and outside of meiotic prophase will provide important and generalized insight into the mechanisms that control repair pathway choice and maintain genome stability.

## Supplementary Material

gkad650_Supplemental_File

## Data Availability

The data that support the findings of this study are contained within the article and the supporting information. All source data generated for this study are available from the corresponding author (Stephen Gray; stephen.gray@nottingham.ac.uk) upon reasonable request.
