# Supplementary material for: Recombinase-independent chromosomal rearrangements between dispersed inverted repeats in Saccharomyces cerevisiae meiosis
Source: Nucleic Acids Res. 2023 Aug 7;51(18):9703–15. doi: 10.1093/nar/gkad650 (PMC10570019; doi:10.1093/nar/gkad650)
Supplement: gkad650_Supplemental_File [file gkad650_supplemental_file.pdf]

Supplemental Figure 1: Ectopic recombinants 1 and 3 are dependent upon *leu2::hisG*

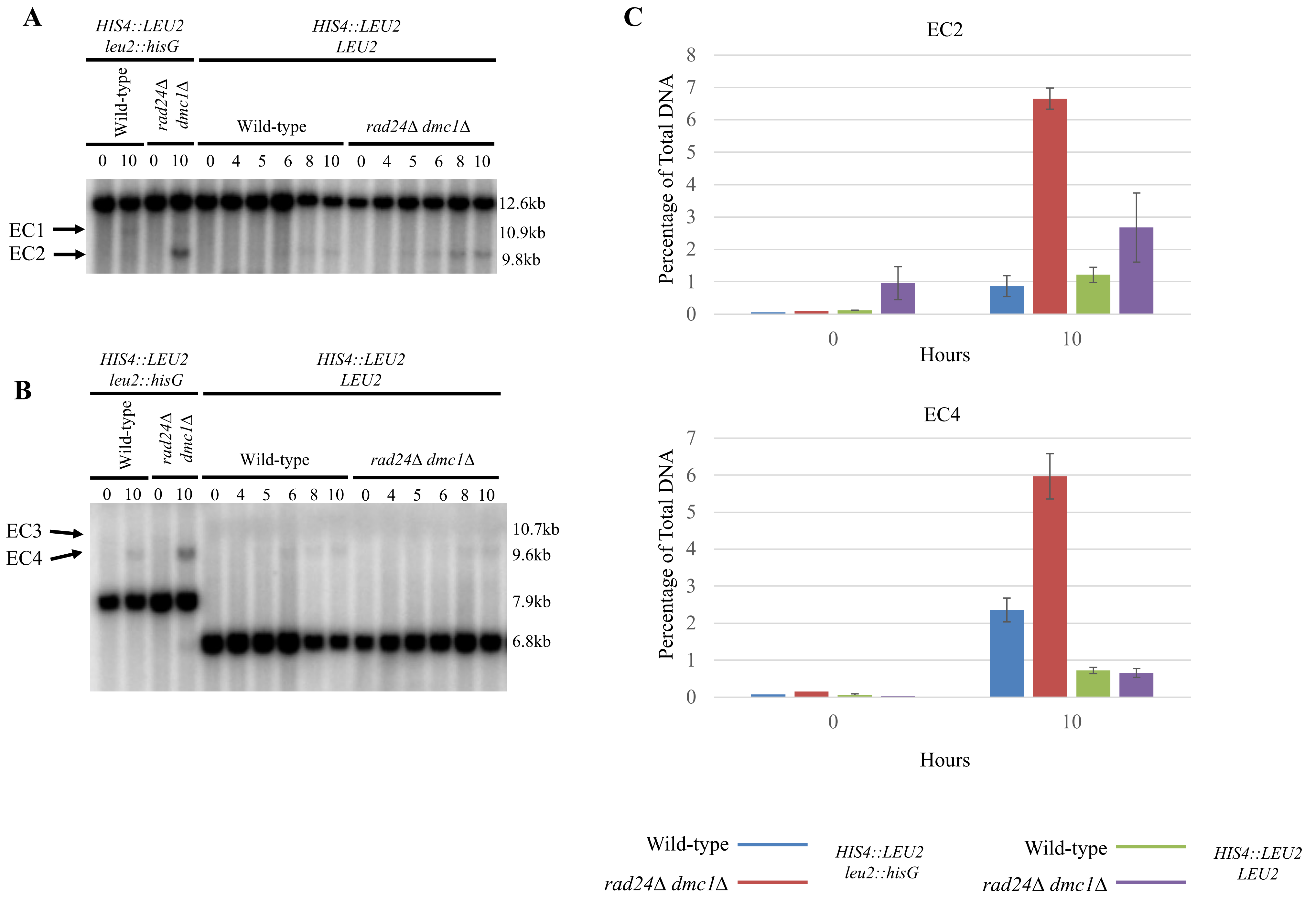

Supplemental Figure 1: Ectopic recombinants 1 and 3 are dependent upon *leu2::hisG*. (A) (A) Southern blot using *HIS4 LH* probe (probe A) and (B) *LEU2* probe (probe B) showing ectopic recombinants (arrows). (C) Quantification of ectopic recombinant levels from (A) and (B) with average ectopic recombinants signal plotted with standard deviation.

Supplemental Figure 2: Ectopic recombinants can form between *HIS4::LEU2* and *nuc1::LEU2*

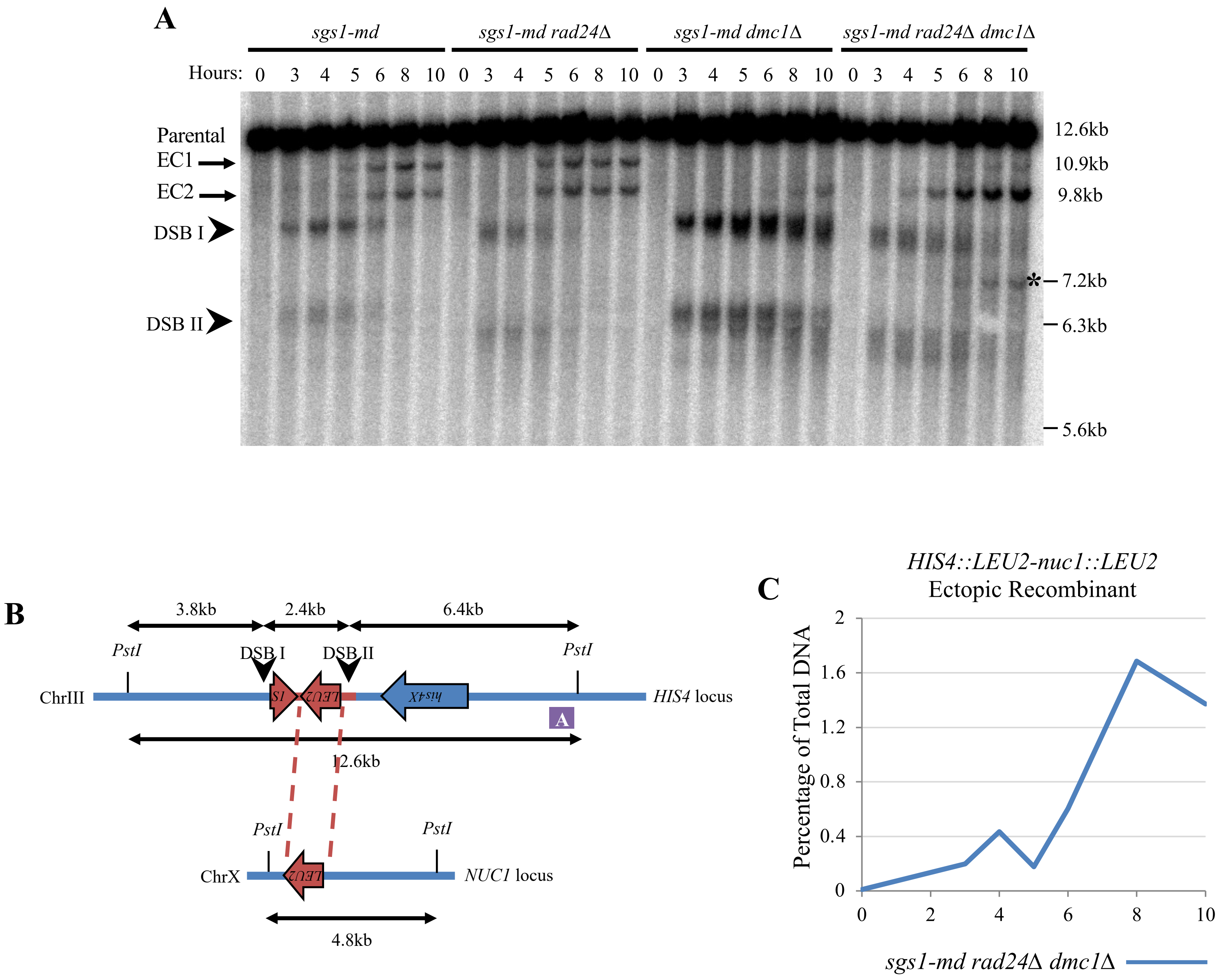

Supplemental Figure 2: Ectopic recombinants can form between *HIS4::LEU2* and *nuc1::LEU2*. (A) Southern blot shown in figure 2 using *HIS4 LH* probe (probe A, purple box), with DSBs and ectopic recombinants. Asterisk indicates additional ectopic recombinant product. (B) Schematic of the *HIS4::LEU2* hotspot on chromosome III (top) and the *NUC1::LEU2* locus on chromosome X (bottom). Dotted lines indicate homology. (C) Quantification of ectopic recombinant shown in (A).

Supplemental Table 1: Summary of mutant phenotypes

Phenotype / Genotype	Recombinant Types	Average Total Ectopic Recombinants (10 hours)	DSB Resection	Checkpoint Function	Inter-homologue Bias	Ectopic Repair Pathways
Wild-type	EC1, EC2, EC3, EC4	5.48%	Normal	Active	Active	HR, RD-SSA, RI-SSA, RD-BIR, RI-BIR
rad24Δ	EC1, EC2, EC3, EC4	9.69%	Hyper-resected	Absent	Defective	HR, RD-SSA, RI-SSA, RD-BIR, RI-BIR
dmc1Δ	EC2, EC4	1.96%	Continued Resection	Arrested	Active	(HR), (RD-SSA), (RD-BIR), RI-SSA, RI-BIR
rad24Δ dmc1Δ	EC2, EC4	13.1%	Hyper-resected	Absent	Defective	(HR), (RD-SSA), (RD-BIR), RI-SSA, RI-BIR
sgs1-md	EC1, EC2, EC3, EC4	17.11%	Normal	Active	Defective	HR, RD-SSA, RI-SSA, RD-BIR, RI-BIR
sgs1-md rad24Δ	EC1, EC2, EC3, EC4	21.24%	Hyper-resected	Absent	Defective	HR, RD-SSA, RI-SSA, RD-BIR, RI-BIR
sgs1-md dmc1Δ	EC2, EC4	5.78%	Continued Resection	Arrested	Defective	(HR), (RD-SSA), (RD-BIR), RI-SSA, RI-BIR
sgs1-md rad24Δ dmc1Δ	(EC1), EC2, EC4	16.22%	Hyper-resected	Absent	Defective	(HR), (RD-SSA), (RD-BIR), RI-SSA, RI-BIR
rad51Δ dmc1Δ	EC2, EC4	9.04%	Continued Resection	Arrested	Active	RI-SSA, RI-BIR
rad24Δ rad51Δ dmc1Δ	EC2, EC4	8.01%	Hyper-resected	Absent	Defective	RI-SSA, RI-BIR

Supplemental Table 1: Summary of Mutant Phenotypes: Genotypes of strains investigated in this study with predominant ectopic recombinant types observed. Average ectopic recombinant levels from figures 1, 2 and 5 at 10 hours. Resection phenotypes as described in (36,37,40,41). Checkpoint function as described from (36,37). Inter-homologue bias as described in this study and (18,24). Potential ectopic recombinant repair pathways with brackets indicating unknown function.

Supplemental Table 2: Genotypes of strains used in this study

Strain	Number	Genotype
wildtype	MJ6	*ho::LYS2/', lys2/', ura3/', arg4-nsp/', leu2::hisG/', his4X::LEU2/', nuc1::LEU2/'*
sae2Δ	MJ315	*ho::LYS2/', lys2/', ura3/', arg4-nsp/', leu2::hisG/', his4X::LEU2/', nuc1::LEU2/', sae2Δ::KanMX/'*
rad24Δ	SG150	*ho::LYS2/', lys2/', ura3/', arg4-nsp/', leu2::hisG/', his4X::LEU2/', nuc1::LEU2/', rad24Δ::hpHMX4/'*
dmc1Δ	SG147	*ho::LYS2/', lys2/', ura3/', arg4-nsp/', leu2::hisG/', his4X::LEU2/', nuc1::LEU2/', dmc1Δ::LEU2/'*
rad24Δ dmc1Δ	SG146	*ho::LYS2/', lys2/', ura3/', arg4-nsp/', leu2::hisG/', his4X::LEU2/', nuc1::LEU2/', dmc1Δ::LEU2/', rad24Δ::hpHMX4/'*
wildtype	MJ1395	*ho::LYS2/', lys2/', ura3/', arg4-nsp/', his4X::LEU2/'*
rad24Δ dmc1Δ	MJ1398	*ho::LYS2/', lys2/', ura3/', arg4-nsp/', his4X::LEU2/', dmc1Δ::LEU2/', rad24Δ::hpHMX4/'*
sgs1-md	SG193	*ho::LYS2/', lys2/', ura3/', arg4-nsp-?/', leu2::hisG/', his4X::LEU2/', nuc1::LEU2/', natMX::pCLB2::3HA::SGS1/'*
sgs1-md rad24Δ	SG153	*ho::LYS2/', lys2/', ura3/', arg4-nsp-?/', leu2::hisG/', his4X::LEU2/', nuc1::LEU2/', rad24Δ::hpHMX4/', natMX::pCLB2::3HA::SGS1/'*
sgs1-md dmc1Δ	SG547	*ho::LYS2/', lys2/', ura3/', arg4-nsp/', leu2::hisG/', his4X::LEU2/', nuc1::LEU2/', natMX::pCLB2::3HA::SGS1/', dmc1Δ::LEU2/'*
sgs1-md rad24Δ dmc1Δ	SG223	*ho::LYS2/', lys2/', ura3/', arg4-nsp-?/', leu2::hisG/', his4X::LEU2/', nuc1::LEU2/', dmc1Δ::LEU2/', rad24Δ::hpHMX4/', natMX::pCLB2::3HA::SGS1/'*
rad51Δ dmc1Δ	SG271	*ho::LYS2/', lys2/', ura3/', arg4-?/', leu2::hisG/', his4X::LEU2/', nuc1::LEU2/', rad51Δ::hisG-URA3-hisG/', dmc1Δ::HphMX/'*
rad24Δ rad51Δ dmc1Δ	SG243	*ho::hisG/', lys2/', ura3/', arg4-nsp/', leu2::hisG/', his4X::LEU2/', nuc1::LEU2/', dmc1Δ::LEU2/', rad24Δ::hpHMX4/', rad51Δ::hisG-URA3-hisG/'*
PRD1::pMN164/PRD1	MJ1140	*ho::LYS2/', lys2/', ura3/', arg4-nsp/', leu2::hisG/', his4X::LEU2/', nuc1::LEU2/', PRD1::pMN164-URA3/PRD1*
PRD1::pMN164/PRD1 rad24Δ	MJ1143	*ho::LYS2/', lys2/', ura3/', arg4-nsp/', leu2::hisG/', his4X::LEU2/', nuc1::LEU2/', PRD1::pMN164-URA3/PRD1, rad24Δ::Hyg/'*
PRD1::pMN164/PRD1 sgs1-md	MJ1146	*ho::LYS2/', lys2/', ura3/', arg4-nsp/', leu2::hisG/', his4X::LEU2/', nuc1::LEU2/', PRD1::pMN164-URA3/PRD1, natMX::pCLB2::3HA::SGS1/'*
PRD1::pMN164/PRD1 sgs1-md rad24Δ	MJ1149	*ho::LYS2/', lys2/', ura3/', arg4-nsp/', leu2::hisG/', his4X::LEU2/', nuc1::LEU2/', PRD1::pMN164-URA3/PRD1, rad24Δ::Hyg/',natMX::pCLB2::3HA::SGS1/'*
